# The Long-Term Effect of Saline and Phosphate Buffer Solution on MTA: An SEM and EPMA Investigation

**Published:** 2007-10-02

**Authors:** Masoud Parirokh, Saeed Asgary, Mohammad Jafar Eghbal, Jamileh Ghoddusi, Frank Brink, Sara Askarifar, Mahmoud Torabinejad, Maryam Raoof

**Affiliations:** 1 *Department of Endodontics, Dental School, Kerman University of Medical Sciences, Kerman, Iran*; 2 *Department of Endodontics, Iranian Center for Endodontic Research, Dental Research Center, Shahid Beheshti University of Medical Sciences, Tehran, Iran*; 3 *Department of Endodontics, Dental School, Mashad University of Medical Sciences, Mashad, Iran*; 4 *Senior Technical Officer, Electron Microscopy Unit, Research School of Biological Sciences, Australian National University, Canberra, Australia*; 5 *Department of Endodontics, School of Dentistry, Loma Linda University, USA*

**Keywords:** EPMA, MTA, PBS, Retro Filling Material, SEM, Storage Media

## Introduction

**INTRODUCTION:** To examine the long-term effects of normal saline and a synthetic tissue fluid (phosphate buffered saline, PBS) on mineral trioxide aggregate (MTA).

**MATERIALS AND METHODS:** Root-ends of twelve extracted human teeth were resected; root-end cavities were prepared and filled with MTA. Samples were randomly divided into two groups of six each. Teeth in group I were placed in normal saline, whilst teeth in group II were placed in PBS. After five months, elemental analysis of the surface of the root-end filling was performed using electron probe microanalysis (EPMA).

**RESULTS:** Results showed that all teeth kept in PBS formed crystal deposits. In contrast, no such crystal formation was observed in teeth kept in normal saline solution. The results of elemental analysis showed that the composition of the crystals observed for teeth kept in PBS was consistent with that of a mineral hydroxyapatite, Ca_10_(PO_4_)_6_(OH)_2_.

**CONCLUSION:** Based on these *in vitro* results, we suggest that the hydration of MTA surface and the release of calcium from MTA in contact with phosphorous of PBS produced hydroxyapatite crystals over MTA and it may be a mechanism which is responsible for cementum formation during *in vivo* studies.

## INTRODUCTION

Healing after periradicular surgery necessitates the regeneration of the apical attachment apparatus, as well as osseous repair of medullary and cortical bone. Deposition of cementum over the resected root end is an essential step in dento-alveolar healing (1). A number of studies have attempted to identify the ideal root-end filling material (2-5). These materials include: amalgam, gutta-percha, composite resins, glass ionomers and zinc oxide eugenol cements such as IRM and Super EBA (3,4,6). Mineral trioxide aggregate (MTA) has been introduced as the most tissue compatible root-end filling material (5,7). Its biocompatibility (8-12), antimicrobial effect (13,14), sealing ability (2,4,15) and marginal adaptation (16) have been confirmed.

MTA is a powder consisting of fine hydrophilic particles that harden when they come in contact with water (17). Hydration of the powder results in a colloidal gel that solidifies to a hard structure. Characteristics of hardened MTA depend on the size of the particles, the water-to-powder ratio, the temperature and humidity at the application site, as well as the amount of air trapped in the mixture (18). A recently published study showed that grey MTA composed of calcium, silicon, aluminium, iron, bismuth and oxygen (19).

The results of MTA studies in experimental animals indicated that MTA significantly causes less inflammation than amalgam and Super EBA (5,20). More importantly, cementum bridge formation occurs directly over the MTA when this material is used as a root-end filling material (5,7).

The mechanism involved in the cementogenesis of MTA is unclear (21). Sarkar *et al*. (22) used MTA as a root canal filling material and kept their samples in a phosphate buffered saline (PBS). They concluded that Ca from MTA reacts with phosphorus in PBS yielding hydroxyapatite. The same results were obtained by Bozeman *et al.'s* study (23). However, previous research studies that kept MTA in different liquids used MTA as a root filling material (22) or placed the material in chlorinated polyvinyl chloride (CPVC) pipe as a mold (23).

One of the characteristics of bioactive materials is the ability to form an apatite-like layer on their surfaces *in-vivo* in contact with physiological fluids (24), or *in-vitro *in contact with simulated body fluids such as PBS (25).

Therefore, the purpose of this study was to compare the chemical reaction occurring on MTA surfaces in contact with normal saline or PBS in simulated clinical conditions.

## MATERIALS AND METHODS

Both the Ethic Human Committees of Kerman University of Medical Sciences and The Australian National University approved this study.

Twelve freshly extracted human single-rooted teeth that were extracted due to either periodontal problems or for other orthodontic reasons were used in this study. After access cavity preparation and root canal preparation by crown down technique, the canals were obturated with gutta-percha (Ariadent, Tehran, Iran) and AH26 (Dentsply, GmbH, Konstanz, Germany) root canal sealer. The apical third of each root was resected using a No.701 fissure bur (Diatech, Heerbrugg, Switzerland) in a high-speed handpiece with copious water spray. In each tooth, a root-end cavity was prepared using an ultrasonic device (EMS-Switzerland). The cavity that was prepared according to the manufacturer’s instruction was filled with grey MTA (ProRoot MTA, Dentsply Tulsa Dental, Tulsa, OK, USA). The teeth were randomly divided into two groups of six each. Teeth in group I were placed in phosphate buffered saline (pH=7.4), whilst teeth in group II were placed in normal saline. The teeth in both groups were incubated at 37°C. After five months, all samples were removed from the solution and air-dried. Each sample was photographed using a stereomicroscope (Wild Heerbrugg-Leica, Switzerland) at ×6.4 to ×32 magnification.

All samples except one in each PBS or normal saline group were used for scanning electron microscopy (SEM) and for elemental analysis. They were coated with ~20nm of carbon. Images were collected using JEOL JSM6400 (JEOL, Japan) and Cambridge S360 (England) SEMs. Energy dispersive x-ray analysis (EDXA) for elemental analyses was carried out on a JEOL 6400 equipped with an Oxford Instruments (Cambridge, England) light element-sensitive detector.

One sample from each PBS and normal saline groups were longitudinally split for observing the relation of the crystals (if present) and MTA.

## RESULTS

**Figure 1:**
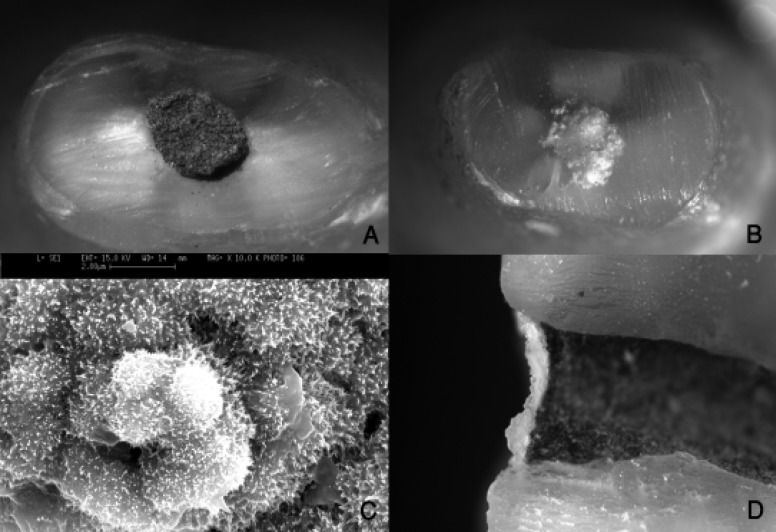
A) MTA surface kept in normal saline for 5 months, B) MTA surface kept in PBS for 5 months, C) Scanning electron micrograph of hydroxyapatite crystal over MTA (×10000), and D) The relationship between MTA and HA crystals from the profile view of a spilt root.

All root-end cavities that were kept in PBS showed a growth of white crystals over the margin of the MTA and resected root ends and in many of them a white plaque over the entire surface of the retrograde cavity could be observed. In contrast, none of the teeth that were kept in normal saline revealed such a pattern of crystal growth over the MTA (Figure 1A),(Figure 1B),(Figure 1C). The sample of PBS group which was split longitudinally showed a thin layer of the crystals formed on MTA (Figure 1D).

**Figure 2:**
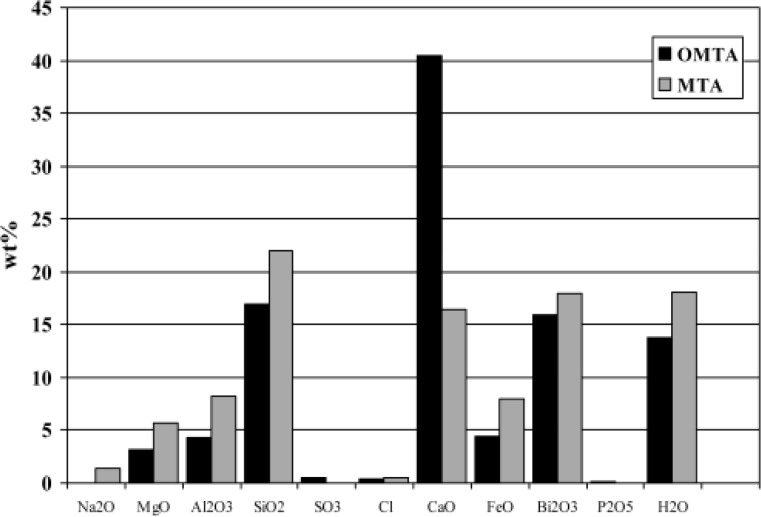
Comparison of electron probe microanalysis between freshly mixed MTA and MTA kept in normal saline for 5 months. For useful comparison, the data has been normalized to the same silicon composition.

**Figure 3:**
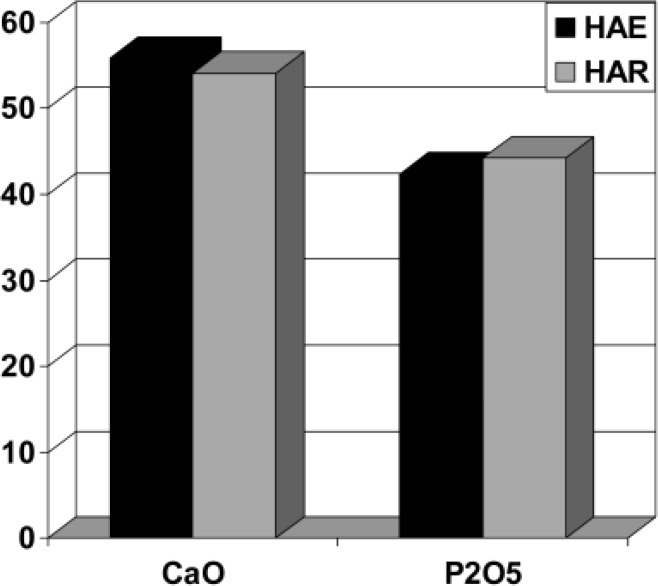
Comparison between compositions of crystals forming over MTA kept in PBS (MTAHA) and the composition of a standard mineral HA (SHA).

Electron probe analysis showed that the MTA kept in contact with saline suffered a significant depletion of calcium (measured as CaO wt% concentration) compared to the original composition of grey MTA (Table 1),(Figure 2). Analyses of the white crystals formed on the MTA kept in PBS indicated formation of a substance with a composition consistent with that of hydroxyapatite (HA, Ca_10_(PO_4_)_6_(OH)_2_) (Table 1),(Figure 3).

## DISCUSSION

Functioning periradicular tissues consist of healthy cementum, periodontal ligament and bone. The ability to enhance the regeneration of the periodontal attachment apparatus is a desirable property for any material used as root canal filling, apexification, root-end filling or perforation repair. Ideally, any material used in these situations should result in not just the formation of new bone, but also periodontal ligament and cementum. Histological reports have indicated that new cementum may be formed adjacent to a few dental materials when placed in contact with periodontal tissues (26). Torabinejad *et al.* believe that the deposition of cementum against MTA may be due to a number of factors, such as sealing ability, biocompatibility or alkaline pH on setting (27).

Research studies have claimed that calcite crystals are formed over calcium hydroxide in contact with pulp tissue, or that the culture medium produces deposition of calcite crystals (28). The same crystals observed with calcium hydroxide were reported for MTA (8). Baek *et al.* in their study found two different calcified material deposits over MTA, a crystalline structure and newly deposited cementum (5). However, recent published studies, as well as the results in this study, have shown that HA crystals are formed over MTA in a tissue-synthetic fluid (*i.e.,* PBS) (22,23).

Sakar *et* *al.* suggest that the two clinically significant properties of MTA, sealing ability and biocompatibility, stem from physico- chemical reactions (22). Previous research studies as well as this study lead us to surmise that the product of this reaction of MTA with the oral environment is not calcite, but HA (22,23). Previous research studies showed that gel glasses containing CaO–P2O5–SiO2 after soaking in a synthetic tissue fluid such as PBS could produce an amorphous layer of calcium phosphate and after 7 days on the surface of the gel glasses needle-like apatite crystallites were appeared (29,30). The results of this study have shown that although MTA has not had phosphorous in its contents the reaction between PBS and MTA surface produced HA. HA exhibits excellent biocompatibility manifested in minimal tissue toxicity and foreign-body reaction, osteoinductivity, as well as osteogenicity (31). The similarity in the mode of biologic action of calcium-containing materials stems from their propensity to release Ca and their ability to form HA.

In this study, the comparison of the measured elemental concentrations of MTA kept in normal saline for five months and the original MTA composition (32) indicated a significant drop in calcium (reported as CaO), while other major elemental constituents appear relatively unchanged (Figure 2). A significant proportion of calcium has been taken up in the solution. Calcium depletion is in agreement with results of previously published studies which have shown that MTA could solubilize and release calcium in contact with aqueous environment (21-23). Previous published studies which used showed that calcium hydroxide was formed (18,19). MTA does not have calcium hydroxide, distilled water as a media for keeping MTA but it has calcium rich phase fraction that could react with tissue fluids to form calcium hydroxide. It has been confirmed that the soluble fraction released from MTA was mainly composed by calcium hydroxide (21).

**Table 1:** Quantitative analysis results (in wt %) for MTA kept in normal saline (NS) and crystals formed on MTA under phosphate buffered saline (PBS) solution. The original MTA (top) and hydroxyapatite (HA) standard mineral composition (bottom) have been included for comparison.

**Composition**	**Na**_2_ **O**	**MgO**	**Al**_2_ **O**_3_	**SiO**_2_	**P**_2_ **O**_5_	**SO**_3_	**Cl**	**CaO**	**FeO**	**Bi**_2_ **O**_3_	**H**_2_ **O**
**Samples **											
MTA	0	3.1	4.3	17	0.18	0.5	0.4	40.45	4.4	15.9	13.7
MTA-NS	1.4 ±0.5	5.7 ±1.3	8.2 ±2.4	22 ±5.0	0	0.3 ±0.4	0.5 ±0.2	16.4 ±3.6	8.0 ±1.1	18.5 ±3.4	18.1 ±3.4
MTA- PBS	-	-	-	-	44.7 ±3.8	-	-	53.5 ±5.8	-	-	1.8
HA	-	-	-	-	42.39	-	-	55.38	-	-	1.70

Two main mechanisms have been described for MTA sealing ability and formation of hard tissue over the material when used as root-end filling, pulp capping materials, and sealing the perforations. Frildand and Rosado and Camilleri *et al.* studies showed that substance released by MTA which were kept in distilled water is basically calcium hydroxide (18,19). On the other hand, when MTA kept in PBS for period of times hydroxyapatite crystals were formed over MTA in two other research studies (22,23). It has been shown that the formation of hard tissue needs high pH which calcium hydroxide production of MTA may help at the first step of MTA placement. Camilleri *et al.* in their experiment observed phosphorous in analysis of set MTA when it had been immersed in phosphorous solution.

Meanwhile, SEM images of above mentioned samples in their study showed crystal formation over MTA (19). Based on the results of this study as well as previously published studies (18,19,21-23) we suggest that hydration of MTA in contact with tissue fluid provide calcium hydroxide which dissociate into calcium and hydroxyl ion. The calcium ion would react with phosphorous of the tissue fluid providing hydroxyapatite over MTA surface which is responsible for MTA advantageous properties that MTA has in contact with dental and periapical tissues. Release of calcium content, production of calcium hydroxide, increasing pH and at last the formation of hydroxyapatite all may be responsible of MTA biocompatibility and sealing ability and bioactive regeneration.

Bozeman *et al.*, in a recently published study reported that the composition of crystals forming in PBS was silicon, calcium, and phosphorus (23). However, in this study, none of the crystals showed silicon on their surface. The distinction between the methods of the two studies may explain that difference.

Baek *et al.* in their study have shown that there were two types of surface reactions over MTA: a crystalline structure, and newly deposited cementum that started mostly from the adjacent dentin, but was sometimes also found in islands and finally appeared as mineralized cellular cementum (5). In this study, all samples showed HA crystals at the rim; however, many of our samples had HA plaque on the resected root ends. Since hydroxyapatite crystals are formed at the margin of the cavity, the deposition of cementum is mostly formed from the adjacent dentin.

Friedland and Rosado observed that set MTA, as with many biomedical cement materials, contains many voids in the form of air bubbles, pores and capillary channels. They pose that the ability of MTA to effectively fill these voids, and other factors including the alkaline nature of MTA, may diminish bacterial leakage (18). However, no previous studies on the sealing ability of MTA (2-4,33-35) had used PBS as a medium, and therefore, no hydroxyapatite were produced on the MTA used as root-end filling material in those experiments.

## CONCLUSION

On the limitations of this *in vitro* study it seems that calcium ions of MTA in contact with phosphorous ions of phosphate buffered saline solution produce hydroxyapatite crystals over MTA and it may be a basis for excellent biocompatibility of this material.
